# Facile electrosynthesis of silicon carbide nanowires from silica/carbon precursors in molten salt

**DOI:** 10.1038/s41598-017-10587-5

**Published:** 2017-08-30

**Authors:** Xingli Zou, Li Ji, Xionggang Lu, Zhongfu Zhou

**Affiliations:** 1 State Key Laboratory of Advanced Special Steel & Shanghai Key Laboratory of Advanced Ferrometallurgy & School of Materials Science and Engineering, Shanghai University, Shanghai, 200072 China; 2 Center for Electrochemistry, Department of Chemistry, The University of Texas at Austin, Austin, Texas 78712 USA; 3 Microelectronics Research Center, Department of Electrical and Computer Engineering, The University of Texas at Austin, Austin, Texas 78712 USA; 4 Institute of Mathematics and Physics, Aberystwyth University, Aberystwyth, SY23 3BZ UK

## Abstract

Silicon carbide nanowires (SiC NWs) have attracted intensive attention in recent years due to their outstanding performances in many applications. A large-scale and facile production of SiC NWs is critical to its successful application. Here, we report a simple method for the production of SiC NWs from inexpensive and abundantly available silica/carbon (SiO_2_/C) precursors in molten calcium chloride. The solid-to-solid electroreduction and dissolution-electrodeposition mechanisms can easily lead to the formation of homogenous SiC NWs. This template/catalyst-free approach greatly simplifies the synthesis procedure compared to conventional methods. This general strategy opens a direct electrochemical route for the conversion of SiO_2_/C into SiC NWs, and may also have implications for the electrosynthesis of other micro/nanostructured metal carbides/composites from metal oxides/carbon precursors.

## Introduction

In recent years, silicon carbide (SiC) nanomaterial has been recognized as a rising star, as demonstrated by an increasing number of published research about it^1–6^. In particular, SiC nanowires (NWs) have attracted intensive attention due to their outstanding performances in many applications, such as power electronics, hostile-environment electronics and sensors^1–11^. To date, tremendous efforts have been devoted to produce SiC NWs^12–24^. Conventionally, SiC NWs can be prepared by template synthesis, chemical vapour deposition, magnesiothermic reduction and solid-state method, *etc* ^2–6, 12–24^. Despite the successful synthesis of SiC NWs by using these methods, however, these synthesis procedures typically require the generation of toxic Si-containing vapour, ultra-high purity precursors, template or catalyst^2–6, 19^. Therefore, searching for new simple template/catalyst-free strategy to synthesize SiC NWs is still extremely needed.

Recently, the molten salt electroreduction process has been intensively investigated for the facile production of micro/nanostructured metals/alloys/composites powders^25–39^. In particular, the electroreduction process in molten salt can directly convert solid metal oxides into micro/nanostructured metals/alloys powders and consumes only electrons as reductant^27, 28^. These innovative previous studies^25–39^ offer an attractive promising strategy for the facile electrosynthesis of micro/nanostructured metal carbides in molten salt. In addition, it has been proved that the distinctive dissolution-electrodeposition mechanism for silica in molten salt (silica → silicate ions → silicon) can contribute to the formation of Si nanowire structure^34–38^. which means that it is also promising for the electrosynthesis of SiC NWs in molten salt.

**Figure 1:**
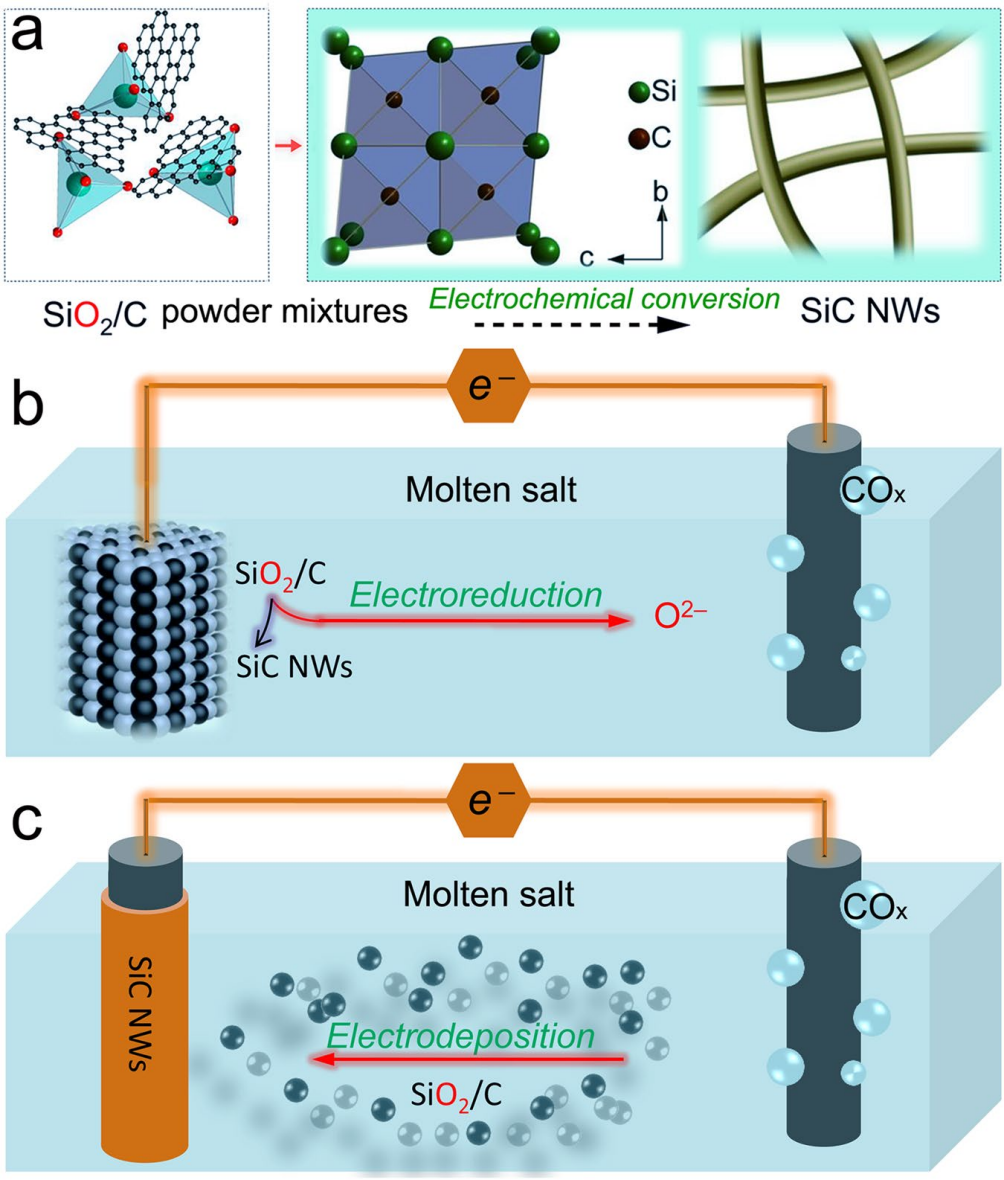
Synthesis strategy of SiC NWs by using a facile molten salt electrosynthesis process. (**a**) Schematic illustration of the direct route from SiO_2_/C to SiC NWs. (**b**) Electroreduction to produce SiC NWs. (**c**) Electrodeposition to produce SiC NWs.

Herein, we report that homogenous SiC NWs can be directly produced from silica/carbon (SiO_2_/C) precursors in molten calcium chloride (CaCl_2_) without using any template and catalyst. The one-step simple route from SiO_2_/C precursors to SiC NWs avoids the requirement of complex procedures, as shown in Fig. 1. Figure 1a schematically shows the electrochemical route from SiO_2_/C precursors to SiC NWs. Figure 1b and c shows that SiC NWs can be produced through electroreduction and/or electrodeposition processes. The attracting feature of this strategy is that only electrons and graphite anode are consumed during the entire synthesis process, and SiO_2_/C precursors can convert directly into SiC NWs. The synthesized SiC NWs show homogenous structure, and the diameters of the nanowires can be well controlled by moderately tuning some experimental parameters. To the best of our knowledge, this is the first report on the electrosynthesis of SiC NWs by such a facile molten salt electroreduction/electrodeposition strategy.

## Results and Discussion

### Electrosynthesis and characterization of SiC NWs

**Figure 2:**
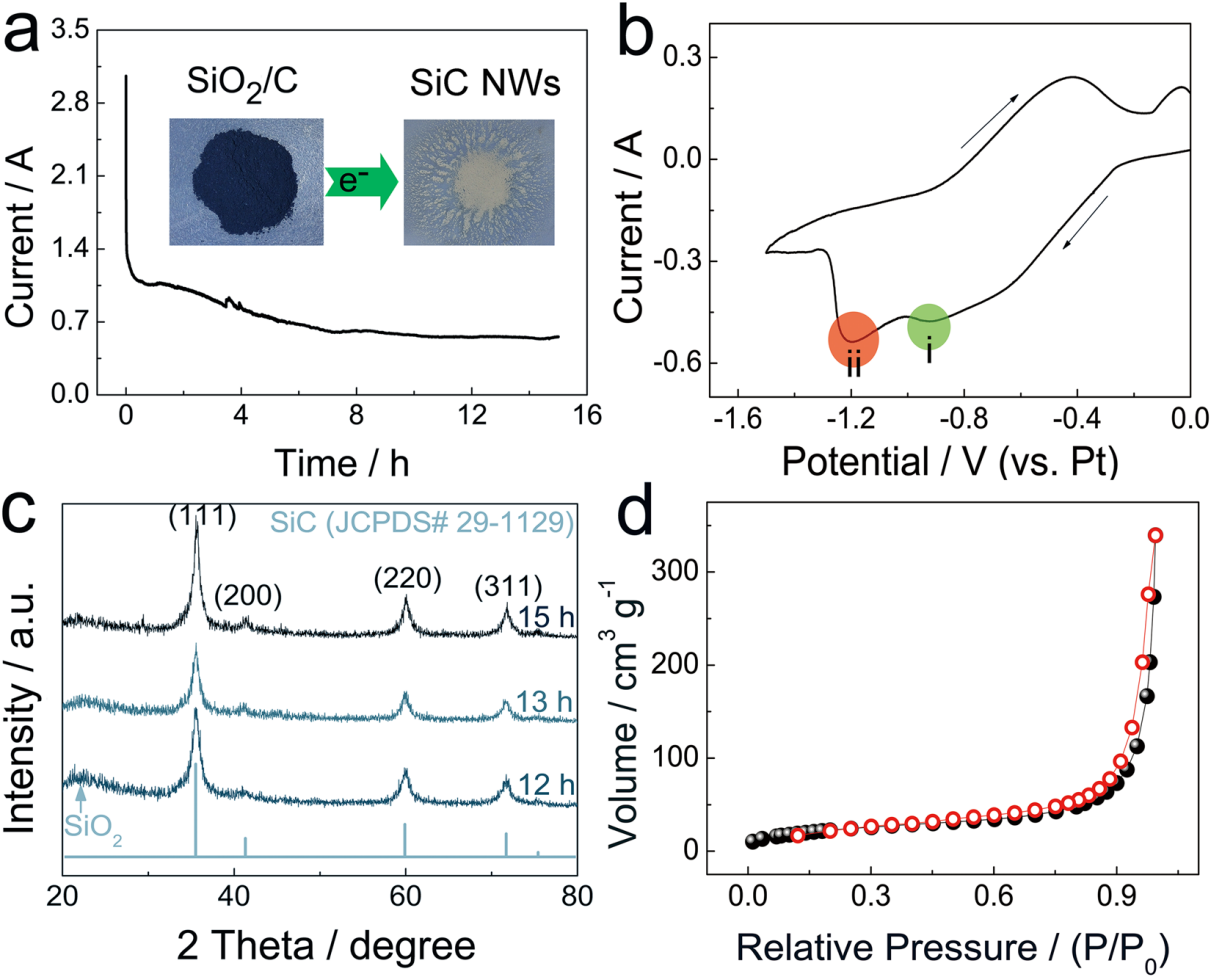
Characterization of the electrosynthesis process and the synthesized SiC NWs product. (**a**) Current-time curve of the electrosynthesis of SiC NWs from SiO_2_/C at 900 °C and 3.1 V in molten CaCl_2_. The insets in (**a**) are the photos of the SiO_2_/C precursors and the synthesized SiC NWs product. (**b**) Cyclic voltammogram of SiO_2_/C in molten CaCl_2_ at 900 °C with a scan rate of 60 mV s^−1^. (**c**) XRD patterns of the synthesized SiC NWs. (**d**) Nitrogen adsorption-desorption isotherm of the synthesized SiC NWs.

Figure 2a shows the current-time curve of the electrosynthesis process from SiO_2_/C to SiC NWs at 900 °C and 3.1 V in molten CaCl_2_. About 2.0 g SiO_2_/C precursors can be completely converted into SiC NWs within 15 h, and the current efficiency is calculated to be approximately 30%. The black SiO_2_/C precursors have been gradually transformed into yellow SiC NWs (Fig. 2a, inset). Cyclic voltammetry (CV) analysis (Fig. 2b) reveals that the electrochemical process typically involves the reduction of SiO_2_ (peak i) and silicates (such as CaSiO_3_ (Ca^2+^, SiO_3_^2−^), peak ii)^35^. This observation is generally consistent with the previous work^34, 35^. It is thus suggested that the reaction mechanism of the reduction of SiO_2_/C in molten CaCl_2_ also contains the solid-to-solid electroreduction (SiO_2_ → Si) and dissolution-electrodeposition (SiO_2_ → SiO_3_^2−^, *etc*., → Si) processes^34, 35^. The X-ray powder diffraction (XRD) patterns of the obtained products exhibit clear diffraction peaks (Fig. 2c), which can be indexed as 3C-SiC phase (JCPDS NO. 29-1129). It also shows that SiO_2_/C can be gradually converted into SiC within 15 h. The nitrogen adsorption-desorption isotherm of the synthesized porous SiC NWs shows that the Brunauer-Emmett-Teller specific surface area (BET SSA) is about 83.50 m^2^ g^−1^ and the Langmuir SSA is approximately 138.35 m^2^ g^−1^ (Fig. 2d).

**Figure 3:**
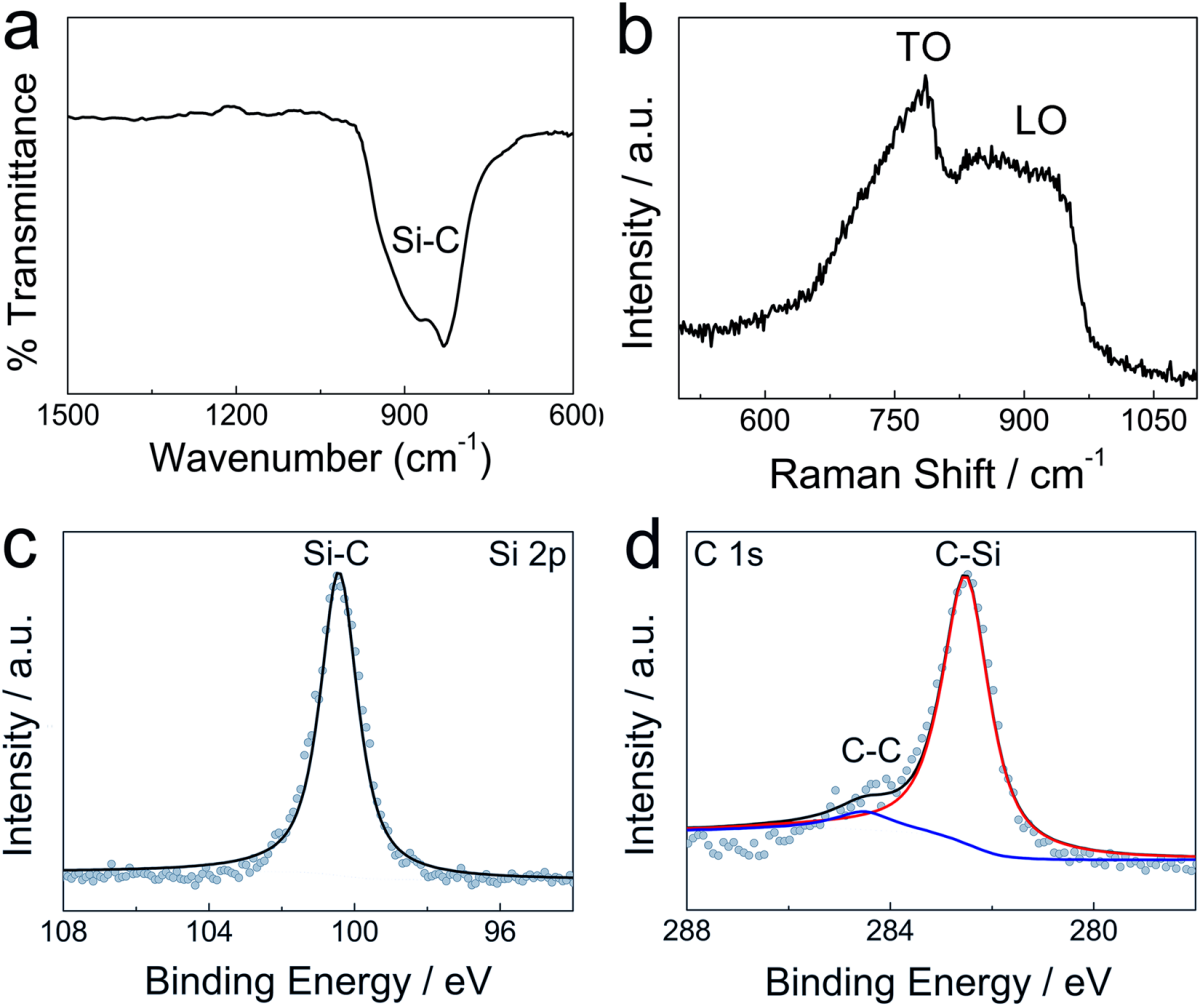
Characterization of the synthesized SiC NWs product. (**a**) FTIR spectrum, (**b**) Raman spectrum and (**c**) (**d**) XPS spectra of the synthesized SiC NWs.

The Fourier transform infrared spectroscopy (FTIR), Raman spectroscopy and X-ray photoelectron spectroscopy (XPS) analyses (Fig. 3) further confirm that the synthesized product is SiC. The FTIR spectrum shows Si-C stretching at approximately 830 cm^−1^ (Fig. 3a). The Raman spectrum (Fig. 3b) of the SiC NWs indicates that the synthesized SiC NWs are similar to the previous work^40^. The XPS spectra of Si 2p and C 1 s regions of the synthesized SiC NWs feature peak maxima at 100.6 eV and 282.5 eV, respectively (Fig. 3c and d), which is the characteristic of SiC^12^. The previous studies^25–39^ generally proved that high-purity metals/alloys/composites powders can be prepared in molten CaCl_2_ from their oxides precursors. In this work, high-purity SiO_2_/C powders were used as precursors, therefore, based on the experimental results (Figs 2c and 3), it is reasonable to believe that the synthesized SiC NWs possess relatively high purity, only a small amount of residual carbon coexists in the product (Fig. 3d).

**Figure 4:**
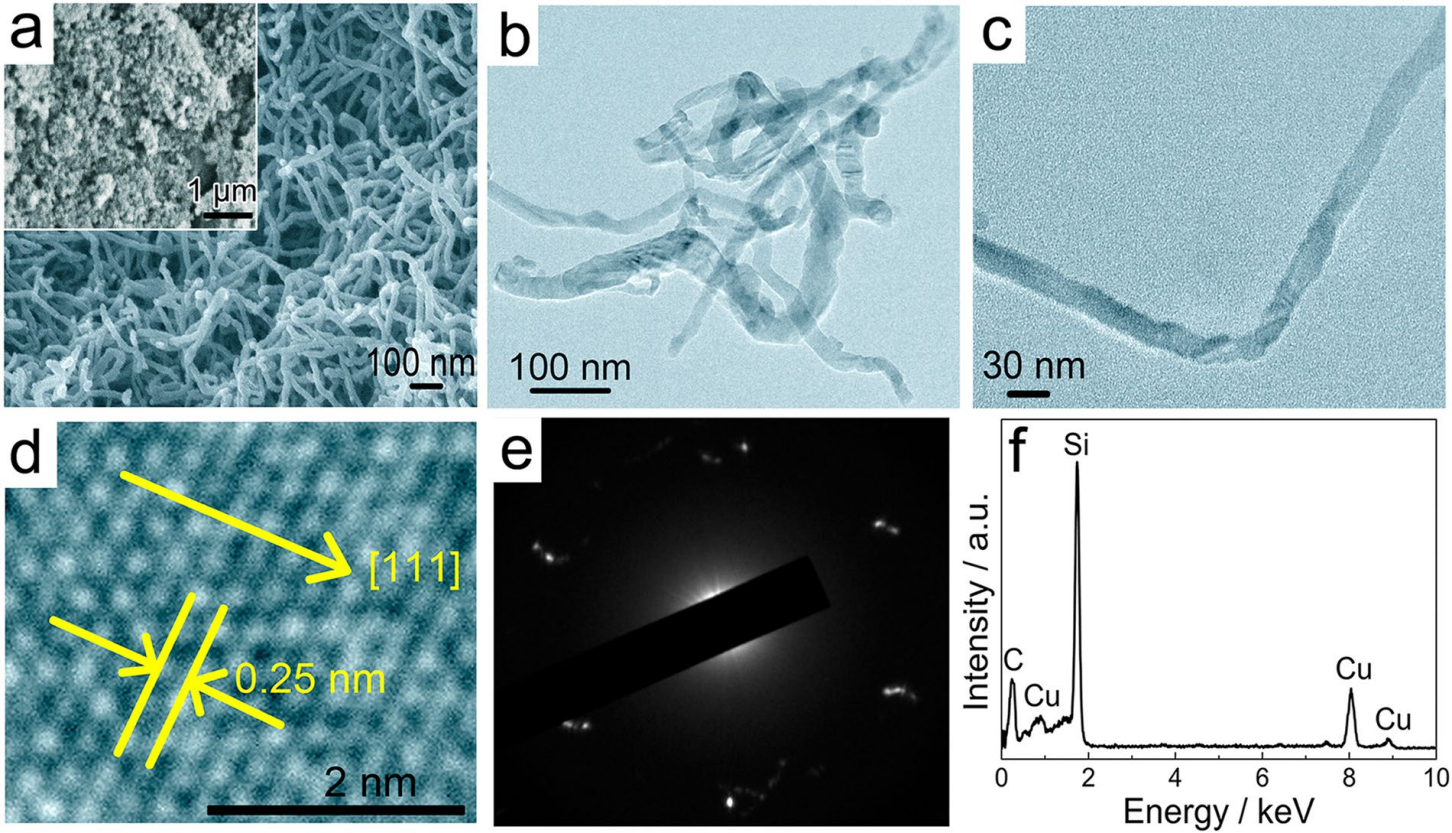
Characterization of the synthesized SiC NWs product. (**a**) SEM image and (**b**) (**c**) TEM images of the synthesized SiC NWs. The inset in (**a**) is the SEM image of the powdered SiO_2_/C precursors. (**d**) HRTEM image and (**e**) its corresponding SAED pattern of the SiC NW shown in (**c**). (**f**) EDS spectrum of the SiC NW shown in (**c**).

The morphology of the synthesized SiC was further characterized by using scanning electron microscopy (SEM) and transmission electron microscopy (TEM), as shown in Fig. 4. Obviously, the mixed powders of SiO_2_/C precursors (Fig. 4a, inset) have been converted into SiC NWs (Fig. 4a). The diameters of the SiC NWs are typically about 30–50 nm. Therefore, it is reasonable to conclude that the homogenous SiC NWs can be produced by using the molten salt electrochemical method without using any template and catalyst. TEM (Fig. 4b and c) and high-resolution TEM (HRTEM) images (Fig. 4d) show that the synthesized SiC NWs are free of any hollow structure. The enlarge HRTEM image and its corresponding selected area electron diffraction (SAED) pattern (Fig. 4d and e) show that the diffraction spots can be indexed based on 3C-SiC crystal structure. The *d* spacing labelled by the parallel yellow lines is measured to be 0.25 nm (Fig. 4d), which is in good agreement with the interplanar spacing of {111} planes of 3C-SiC, implying that the SiC NWs grow along the [111] direction, as shown in Fig. 4d. Energy-dispersive X-ray spectroscopy (EDS) spectrum (Fig. 4f) further confirms that the NWs only consist of Si and C.

### Reaction mechanism and electrodeposition of SiC NWs

Previous studies^34–38^ have successfully converted SiO_2_ into Si NWs in molten CaCl_2_ through the electroreduction process. Based on the observed similar morphology of the synthesized Si NWs and SiC NWs, it is believable that the electroreduction of SiO_2_/C to SiC NWs has a similar reaction mechanism to the electroreduction of SiO_2_ to Si NWs in molten CaCl_2_, *i.e*., the solid-to-solid electroreduction and dissolution-electrodeposition mechanisms^28, 34, 35^. In particular, the compounding process (Eq. (1)), dissolution process (Eq. (2)), electrodeposition process (Eq. (3)) and carbonization process (Si + C → SiC, ∆*G* ^0^ = −63.94 kJ mol^−1^ (900 °C)) may be responsible for the formation of SiC NWs.

1 $$x{{\rm{SiO}}}_{2}+y{{\rm{Ca}}}^{2+}+z{{\rm{O}}}^{2-}\to {{\rm{Ca}}}_{y}{{\rm{Si}}}_{x}{{\rm{O}}}_{(2x+z)}(x=1,2;\,y=1,2,3;\,z=1,2,3)$$

2 $${{\rm{Ca}}}_{y}{{\rm{Si}}}_{x}{{\rm{O}}}_{(2x+z)}\to y{{\rm{Ca}}}^{2+}+{{\rm{Si}}}_{x}{{{\rm{O}}}_{(2x+z)}}^{2y-}$$

3 $${{\rm{Si}}}_{x}{{{\rm{O}}}_{(2x+z)}}^{2y-}+4x{{\rm{e}}}^{-}\to x{\rm{Si}}+(2x+z){{\rm{O}}}^{2-}$$

**Figure 5:**
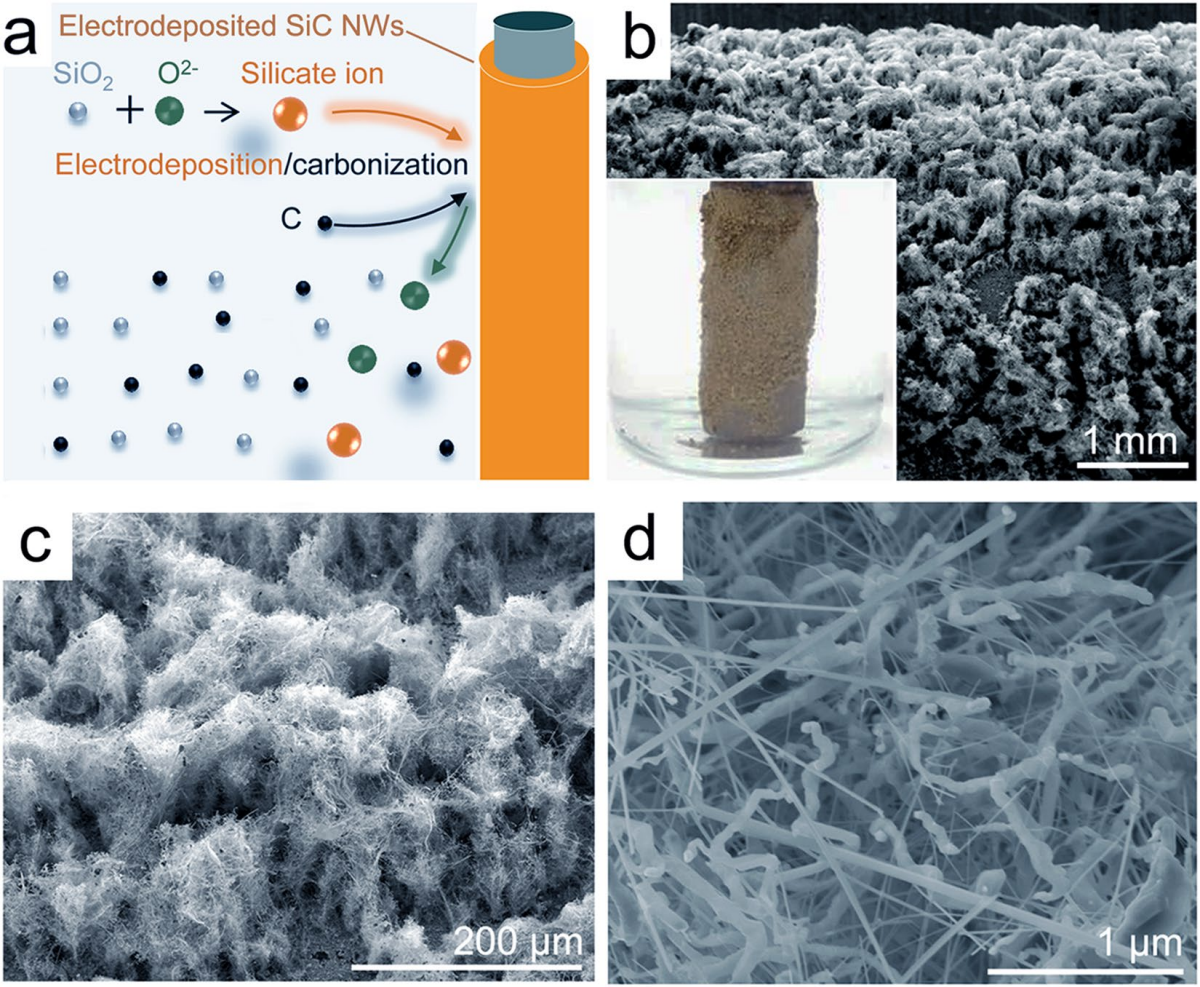
Characterization of the electrodeposited SiC NWs product. (**a**) Schematic illustration of the electrodeposition of SiC NWs from SiO_2_/C powders dispersed in molten CaCl_2_. (**b**–**d**) SEM images of the electrodeposited SiC NWs, the inset in (**b**) is the photo of the SiC NWs electrodeposited on a graphite rod.

To confirm the dissolution-electrodeposition mechanism can contribute to the formation of nanowire structure, new experiment was designed to electrodeposit SiC NWs from SiO_2_/C powders dispersed in molten CaCl_2_. Figure 5a shows the electrodeposition process from SiO_2_/C to SiC NWs through dissolution-electrodeposition processes. Figure 5b–d shows the macro/microstructure of the SiC NWs obtained through the electrodeposition process from SiO_2_/C precursors. The photo of the deposited SiC NWs also exhibits yellow colour (Fig. 5b, inset), which shows almost the same appearance with the SiC NWs obtained through the electroreduction process (Fig. 2a, inset). The SEM images (Fig. 5b–d) of the electrodeposited SiC NWs further confirm that the dissolution-electrodeposition process can lead to the formation of similar nanowire structure. Actually, in our previous work^41^, it is also proved that the dissolution-electrodeposition mechanism in low temperature electrolyte can generate totally different morphologies compare to its precursors.

**Figure 6:**
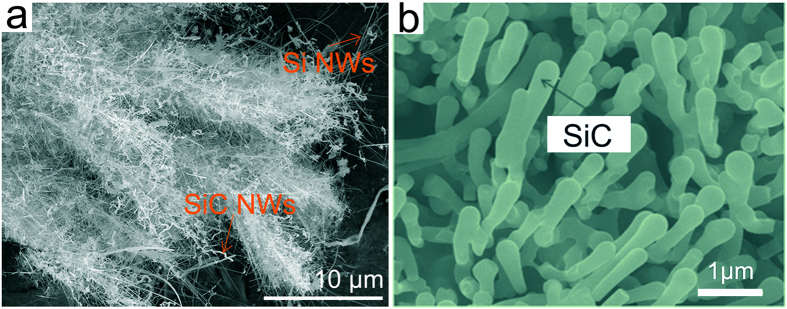
Morphology of the synthesized SiC NWs. (**a**) SEM image of the product electrodeposited from SiO_2_/C powders dispersed in molten CaCl_2_, the product mainly contains SiC NWs and Si NWs. (**b**) SEM image of the SiC NWs electrosynthesized from microscale (~2 μm) SiO_2_/C precursors (electrosynthesis experiment conditions: 900 °C, 3.1 V, 15 h).

It should be noted that the electrodeposition-generated nanostructures typically contain Si NWs and SiC NWs (Fig. 6a), which is mainly caused by the insufficient carbonization process, because the electrodeposition-generated Si nuclei cannot be completely surrounded by the carbon powder dispersed in molten CaCl_2_. This observation suggests that Si NWs can also be prepared through electrodeposition in molten CaCl_2_ from SiO_2_ powder. In addition, the electrodeposition process in molten salt means that the nanowire growth process would not be limited by the space, which is beneficial for the growth of the nanowire to form larger size (includes diameter and length). Besides, the particle size of the precursors (SiO_2_/C) can also influence the morphology of the final product, the SiC NWs with approximately 300 nm (Fig. 6b) can be obtained from the mixed powders of SiO_2_/C with average particle size of approximately 2 μm. It is thus suggested that SiC NWs with different diameters can be synthesized in a controlled manner. Based on the experimental results and previous studies^32–38^, it is reasonable to believe that SiC NWs and Si NWs can be facilely produced by using the molten salt electroreduction and/or electrodeposition processes.

**Figure 7:**
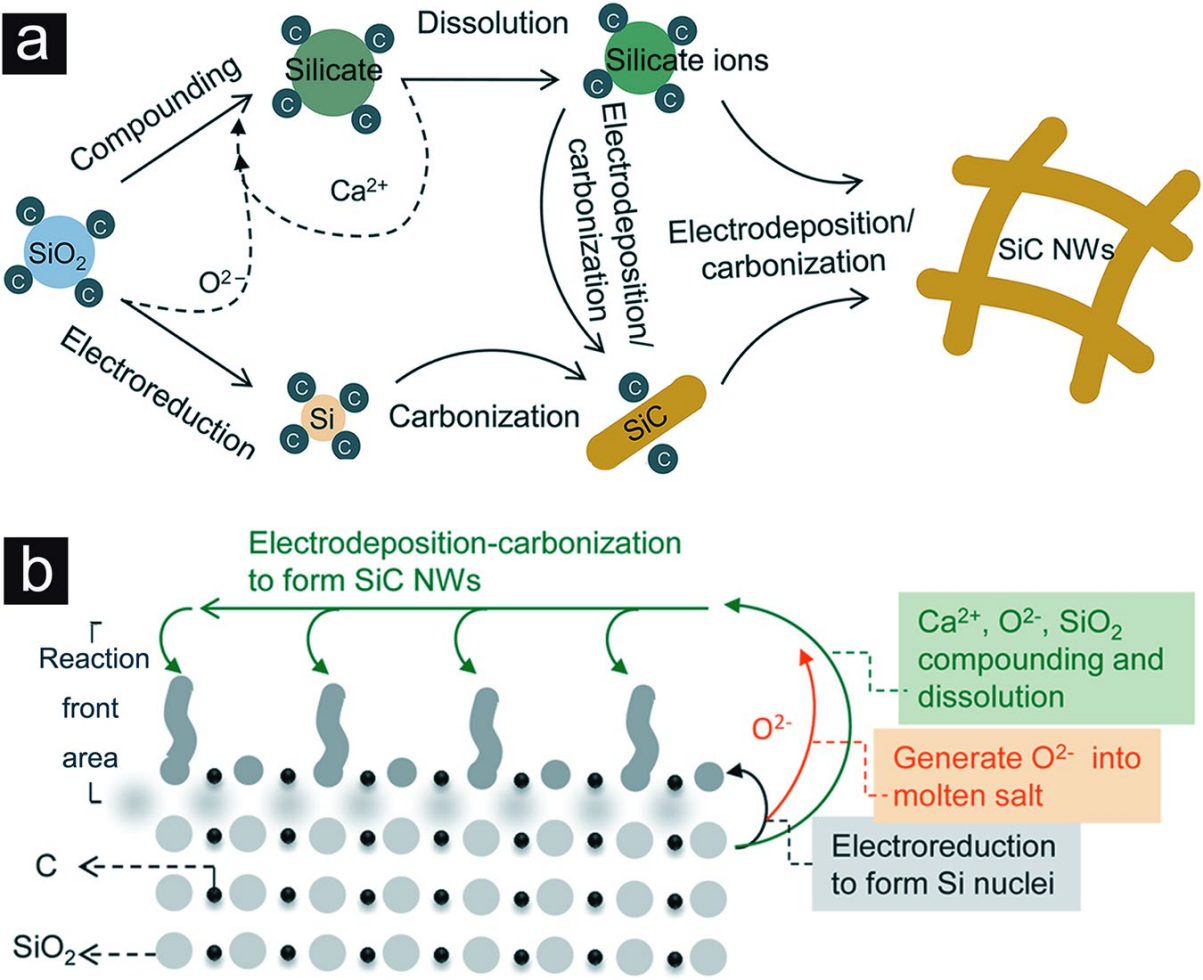
Schematic illustrations of the reaction mechanism. (**a**) The proposed reaction routes from SiO_2_/C to SiC NWs in molten CaCl_2_. (**b**) The formation of SiC NWs from SiO_2_/C precursors, which generally includes the solid electroreduction, compounding-dissolution and electrodeposition-carbonization processes.

Based on the observation in this work and the previous work^32–38^, the reaction mechanism of the molten salt electrosynthesis of SiC NWs from SiO_2_/C can be summarized as two reaction routes, as shown in Fig. 7a, *i.e*.,

(i) Compounding → dissolution → electrodeposition/carbonization → SiC NWs (*i.e*., SiO_2_/C → Ca_*y*_Si_*x*_O_(2*x*+*z*)_/C → Si_*x*_O_(2*x*+*z*)_^2*y*−^/C → Si/C → SiC NWs);

(ii) Solid-to-solid electroreduction → carbonization/electrodeposition → SiC NWs (*i.e*., SiO_2_/C → Si/C/Ca_*y*_Si_*x*_O_(2*x*+*z*)_ → SiC/Si_*x*_O_(2*x*+*z*)_^2*y*−^/C → SiC NWs).

Figure 7b schematically shows the SiC NWs formation process from SiO_2_/C powders in molten salt. This distinct reaction mechanism may lead to the formation of homogenous SiC NWs. Actually, the reaction mechanism in molten salt is usually distinct due to the molten salt electrolyte can provide the microgravity field during the synthesis process, which can contribute to the formation of some special structures, such as nanowire/nanotube and hollow particle^39^. It should be noted that the proposed reaction mechanism may only be used as general guidelines for understanding the reaction mechanism of the electrosynthesis process. Actually, the experimental result suggests that the dissolution-deposition mechanism may mainly occur in the reaction front area (porous spacing area), which may contribute to the formation of nanowire structure during the electroreduction process. However, more details about this assumption need to be investigated, we are continuing our studies to investigate the detailed reaction mechanism of the synthesis process, and further investigation will be reported in due course.

## Conclusions

We introduced a facile method to electrosynthesize SiC NWs from inexpensive and abundantly available SiO_2_/C precursors in molten CaCl_2_. By comparing the electroreduction-produced SiC NWs and the electrodeposition-produced SiC NWs, the dissolution-electrodeposition mechanism has been confirmed to be responsible for the SiC NWs formation. This simple and scalable method shows a great potential to be used for the facile synthesis of uniform SiC NWs, and the diameter of the SiC NWs can be well controlled by moderately tuning some experimental parameters. In addition, it is suggested the electrodeposition process also has the potential to be used for the production of Si NWs. These results may have implications for the synthesis of other micro/nanostructured metal carbides through the molten salt electrochemical process.

## Methods

### Fabrication of the SiO_2_/C cathode and graphite anode

The high-purity homogenous nanoscale (approximately 50–100 nm) and microscale (approximately 2 μm) silica powder and carbon powder were mixed at an accurate stoichiometric molar ratio (SiO_2_:C = 1:1) corresponding to SiC product. Then about 2.0 g SiO_2_/C mixture was pressed under appropriate pressure (8–15 MPa) to form porous SiO_2_/C pellet. The preformed SiO_2_/C pellet, with appropriate open porosity (~28.1%) was sandwiched between two porous nickel foils and attached to electrode wire to from a cathode. The porous nickel foils possess uniform structure (porosity: ~95%, pore per inch: 110, area density: 380 g m^−2^, pore size: 0.2 to 0.4 mm) and can keep stable in molten CaCl_2_^32, 33^, which can be used as the extended electronic conductor to provide more uniform electric contact points to the SiO_2_/C pellet precursors. It is proved that the porous nickel foils almost cannot influence the product in molten salt^31–33^. In addition, a consumable graphite-based anode (high-density graphite rod, *d* = 6 mm) was used for the electrosynthesis of SiC NWs in this experiment.

### Molten salt electroreduction experiment

Molten salt electroreduction experiment was systematically carried out by using the assembled cathode and the graphite-based anode. The electroreduction process employed approximately 150 g anhydrous CaCl_2_ as electrolyte. An alumina crucible served as the electrolytic cell container. The fabricated cathode pellet and the graphite anode were assembled in the alumina crucible to form an electrolytic cell, as shown in Fig. 1. Ultra high purity argon gas was continuously purged through the inside of the electrolytic cell to maintain an inert atmosphere during the entire experimental process. When the system temperature reached the experimental temperature (900 °C), appropriate constant potential (~3.1 V) would be applied between the cathode and the anode system. A Biologic HCP-803 electrochemical workstation was used to record current-time curves during the electroreduction experiment. Oxygen component was gradually electroreduced and migrated through molten salt and then to the graphite anode, where it was oxidized by carbon to form CO/CO_2_ gases. Simultaneously, SiC NWs was electrosynthesized at the cathode after the oxygen contained in the precursors was completely electroreduced (generally needs approximately 15 h). After the electroreduction process, the cathode products were cooled down to ambient temperature and washed through water to remove residual CaCl_2_, and then dried at approximately 100 °C.

### Cyclic voltammetry experiment

CV experiment was carried out in three-electrode electrolytic system, in which a metallic cavity electrode (MCE) with a ~0.5 mm circular hole^29, 30^ filled with SiO_2_/C powders was used as working electrode, a Pt wire and the graphite anode were served as reference electrode and counter electrode, respectively. Before the CV experiment, the electrolytic cell (approximately 100 g anhydrous CaCl_2_) was pre-electrolyzed at 2.5 V for 3–5 h to remove the residual moisture and other redox-active impurities. A Biologic HCP-803 electrochemical workstation was used for the CV experiment. More experimental details related to the CV experiment and the electrochemical reduction experiment can be found in our previous work^31–33^.

### Molten salt electrodeposition experiment

To confirm the dissolution-electrodeposition mechanism of the reaction process, the molten salt electrodeposition experiment was also designed and performed in this work. SiO_2_/C powders were dispersed/dissolved in molten calcium chloride, and then two graphite rods were used as cathode substrate and anode, respectively. The electrodeposition experiment was carried out at 900 °C and 3.1 V for 5–10 h. After the electrodeposition experiment, the cathode graphite rod was taken out and washed through water as well as dried.

### Characterization

The morphology of the electrosynthesized products was characterized using a JEOL JSM-6700F field-emission-type scanning electron microscope (FESEM), and using a JEOL JEM-2010F high-resolution transmission electron microscope (HRTEM) operating at 200 kV. The elemental composition of the samples was analyzed by energy-dispersive X-ray spectroscopy (EDS) attached to the SEM and TEM. Powder X-ray diffraction (XRD) patterns were collected on a Rigaku D/Max-2550 diffractometer using Cu Kα radiation (λ = 0.15406 nm) operated at a voltage of 40 kV and 100 mA. Digital optical photograph of the sample was taken by a KEYENCE VHX-1000C digital optical microscope. X-ray photoelectron spectroscopy (XPS) analysis was performed on an ESCALAB 250Xi spectrometer with Al Kα radiation. Raman spectroscopy of the sample was performed on a Renishaw InVia Raman microspectrometer using an Ar ion laser (514.5 nm). Fourier transform infrared spectroscopy (FTIR) spectrum was recorded on a Nicolet Avatar 380 spectrometer. N_2_ adsorption and desorption isotherm was measured using a Micromeritics ASAP 2020 sorptometer at liquid nitrogen temperature (−196 °C). Before the measurement, the sample was degassed at 200 °C for 6 h. The specific surface area was evaluated using the Brunauer-Emmett-Teller (BET) method.
